# Study of the Effect of *Dillenia indica* Fruit Mucilage on the Properties of Metformin Hydrochloride Loaded Spray Dried Microspheres

**DOI:** 10.1155/2014/628382

**Published:** 2014-11-19

**Authors:** Hemanta Kumar Sharma, Lila Kanta Nath

**Affiliations:** Department of Pharmaceutical Sciences, Dibrugarh University, NH-37, Dibrugarh, Assam 786004, India

## Abstract

Natural materials are preferred over synthetic counterparts because of their biodegradable and biocompatible nature. The present work was proposed to utilize mucilage from natural source for the development of controlled release formulation of metformin hydrochloride. Natural mucilaginous substance extracted from *Dillenia indica* L. (DI) fruit was used in fabricating controlled release microspheres. The microspheres were prepared by spray drying method under different formulation parameters. The prepared microspheres were studied for particle size, drug excipient compatibility, particle shape and surface morphologies, drug entrapment efficiency, mucoadhesivity, and *in vitro* drug release properties. The prepared microspheres exhibited mucoadhesive properties and demonstrated controlled release of metformin hydrochloride. The study reveals that the natural materials can be used for formulation of controlled release microspheres and would provide ample opportunities for further study.

## 1. Introduction

Microspheres can be prepared by different methods. In the present study, microspheres were prepared by spray drying method [1, 2]. Spray drying is advantageous as it is a well-established technology and the equipment is capable of high product output. Thermosensitive substances can be coated successfully by this method because the time of exposure to elevated temperature is extremely short. Nonaqueous coating systems can be used for encapsulation of moisture-sensitive materials. The biological half-life of metformin hydrochloride (MH) is 2.5 to 5 hr and gastrointestinal absorption is incomplete with an absolute bioavailability of 50–60% (under fasting condition). Reduction of gastrointestinal motility enhances the absorption of MH. The rate of absorption is slower than that of elimination.

**Figure 1:**
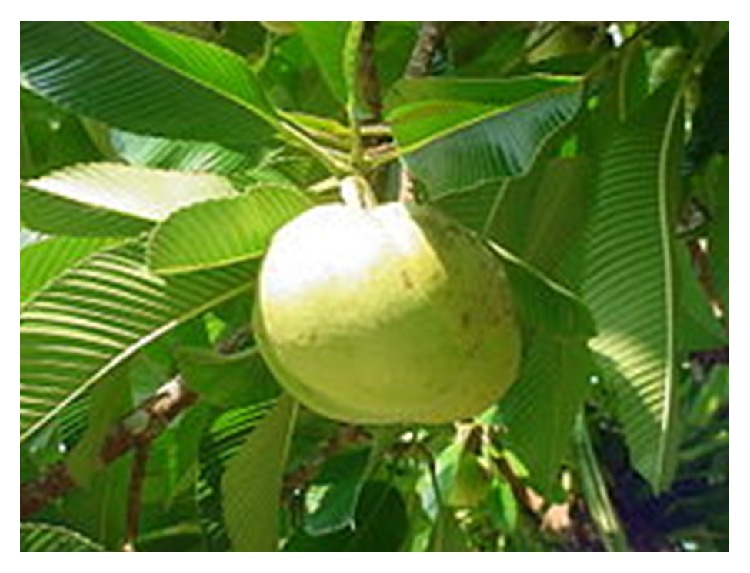
Image of the fruit of* D. indica*.

The plant* Dillenia indica *L. (Family: Dilleniaceae; Synonym:* Dillenia speciosa* Thunb.) is known as elephant apple/indian catmon (English);* Bhavya, Bharija* (Sanskrit);* Ou tenga* (Assamese). It is distributed in India, Nepal, Bangladesh, and Sri Lanka [3]. The fruits of* Dillenia indica* (DI) are used by tribal and folk communities of various regions as vegetable. The fruit of DI is “globose” (Figure 1), with excrescent calyx, 12.5–15.0 cm in diameter, green when young and yellowish and sweet-scented when ripe; seeds many, compressed, embedded in hairy cells [3, 4]. The leaf, bark, and fruit of this plant are used traditionally as medicine in different forms for their therapeutic values. The mucilage of the fruit is used to wash hair and has conditioning effect. Description of* D. indica* is available in various ancient Indian literatures about its important common and medicinal uses [5, 6]. There are also reports that the plant parts exhibited antileukemic, anti-inflammatory, antioxidant, antiproliferative, antidiabetic, antimicrobial, antifungal, antidiarrheal, cytotoxic, and hepatoprotective activities [7–9].

Earlier, extraction, characterization of the mucilage, and compatibility study of the mucilage with metformin hydrochloride was reported [10]. The mucilage was extracted by heating with water at 60–70°C and the filtrate was precipitated by acetone. Quantitative estimation of pectin was carried out after detection of the presence of pectin in the mucilage by colorimetric method. DSC, FTIR, and XRD studies were carried out alone and in combination with metformin hydrochloride. No interaction was found between the mucilage and metformin hydrochloride. Metformin hydrochloride loaded microspheres were prepared using* Bora* rice flour in combination with sodium alginate [11]. In another study, mucilage of DI fruit, mucilage of* Abelmoschus esculentus* pods and Bora rice flour were used for formulation of metformin hydrochloride loaded microspheres [12].

In this study, metformin hydrochloride loaded microspheres were prepared by spray drying method using the mucilage of DI fruits alone. The effect of the mucilage concentration on the properties of the microspheres was studied. The results of this study are reported here.

## 2. Experimental Section

### 2.1. Materials

Metformin hydrochloride (MH) was received as gift sample from Ozone Pharmaceutical Ltd., Guwahati, Assam. The fruits of* Dillenia indica* were procured from local market. The mucilage of* D. indica* fruit was extracted by acetone precipitation method as described above [10, 13]. All chemicals used in this study were of analytical grade and were procured commercially (E-Merck, India). They were used as such without testing and purification. The intestinal segment of goat (Ileum), used for mucoadhesive study, was procured from local slaughter house.

## 3. Methods

### 3.1. Preparation of Microspheres

Metformin hydrochloride loaded microspheres with the mucilage of DI fruits were prepared by spray drying method. Aqueous dispersions of the mucilage of DI fruits of three different concentrations (1.0%, 2.0%, and 3.0% w/v) were prepared by heating at 60 ± 0.5°C for 1 hr. The dispersions were allowed to cool to room temperature and then MH was added to dispersions at a concentration of 1.5% w/v and stirred to dissolve MH. The mixture was sonicated for 10 min. Then, the mixture was spray dried using lab spray dryer (LU-222 Advanced Labultima, India) with 0.7 mm nozzle. Parameters of spray dryer were changed and products were collected separately in each run. The composition and various processing parameters of the method used are presented in Table 1.

### 3.2. Evaluation of Microspheres

The prepared microspheres were evaluated for particle size, particle shape and surface topography, liquid uptake capacity, drug entrapment efficiency, mucoadhesive property, and* in vitro* drug release property.

### 3.3. Particle Size and Shape

The particle size of the microspheres was determined by optical microscopy method [14]. The shape and surface topography of the microspheres was investigated by using (S-3600N, HITACHI) scanning electron microscope (SEM) at 15 KV.

### 3.4. Liquid Uptake Capacity

The liquid uptake capacity of microspheres was determined in three aqueous media (water, 0.1 M HCl and phosphate buffer pH 7.4) at room temperature (37.5°C ± 0.5°C). Weighed quantities of the samples were taken into test tubes separately with fixed volume of media in undisturbed condition for 6 hrs. The liquid was then decanted off carefully and the final weight of microspheres was taken. The percentage liquid uptake was calculated using the following relation:

(1) $$\begin{array}{c} \text{Uptake}\left(\%\right)=\left[\frac{W_{t}-W_{0}}{W_{0}}\right]\times 100, \end{array}$$

where *W*_0_ and *W*_*t*_ are the initial weight and final weight of sample after 6 hrs, respectively [15, 16].

### 3.5. Drug Entrapment Efficiency (DEE)

Microspheres equivalent to 50.0 mg of the drug were taken and the amount of drug entrapped was estimated by dispersing the crushed microspheres in 100.0 mL of phosphate buffer (pH 7.4) by shaking on a mechanical shaker (Remi Motors, Mumbai, India) for 12 hr. The solution was filtered and the filtrate was diluted 25 times with phosphate buffer (pH 7.4) and the absorbance was measured spectrophotometrically (UV-1700, Shimadzu, Japan) at 233 nm against a blank. The amount of drug entrapped in the microspheres was calculated in percentage from the ratio of actual drug content to theoretical drug content as given in the following equation [17, 18]:

(2) Drug  Entrapment  Efficiency%  =Actual  drug  contentTheoretical  drug  content×100.

### 3.6. Mucoadhesive Property

The mucoadhesive property of microspheres was evaluated by* in vitro* wash off test method [19–21]. Freshly excised pieces of goat intestinal mucosa (2 × 2 cm) were washed with phosphate buffer (pH 7.4) mounted on to glass slides with cotton thread. Microspheres were spread on to each prepared glass slide and were counted under microscope. The number of microspheres spread was not constant but ranges from 76 to 98. Then immediately thereafter, the slides were hung to USP tablet disintegration test apparatus (Lab. India, Mumbai). When the test apparatus was operated, the sample was subjected to slow up and down movement in the test fluid (phosphate buffer pH 7.4) at 37°C contained in a 1 L vessel of the apparatus. After 5 hrs, the apparatus was stopped and the number of microspheres still adhering to mucosal surface was counted with the help of microscope. This process was repeated for three times (*n* = 3) and the average number of microspheres adhered was calculated.

### 3.7. *In Vitro* Drug Release Study

The release of drug from the microspheres was studied in phosphate buffer (pH 7.4) as dissolution medium, using USP paddle-type dissolution test apparatus (six vessels, TL 06, Electro Lab, India) at 37 ± 0.5°C with a rotating speed of 100 rpm. The liquid uptake capacity and mucoadhesive property were found to be more in phosphate buffer (pH 7.4) and therefore the* in vitro* drug release study was carried out using phosphate buffer media (pH 7.4). A sample of microspheres equivalent to 50.0 mg of metformin hydrochloride was used in each test. 10.0 mL of the sample from each dissolution test vessel was withdrawn at predetermined time intervals (0, 0.5, 1, 1.5, 2, 4, 6, 8, and 10 hr) for 10 hrs and the same volume of media was replaced. The withdrawn samples were filtered through a membrane filter (0.45 *μ*m) and were analyzed for drug content spectrophotometrically at 233 nm using the UV-visible spectrophotometer (UV-1700, Shimadzu, Japan) [22–24].

## 4. Results and Discussion

### 4.1. Particle Size and Shape

**Figure 2:**
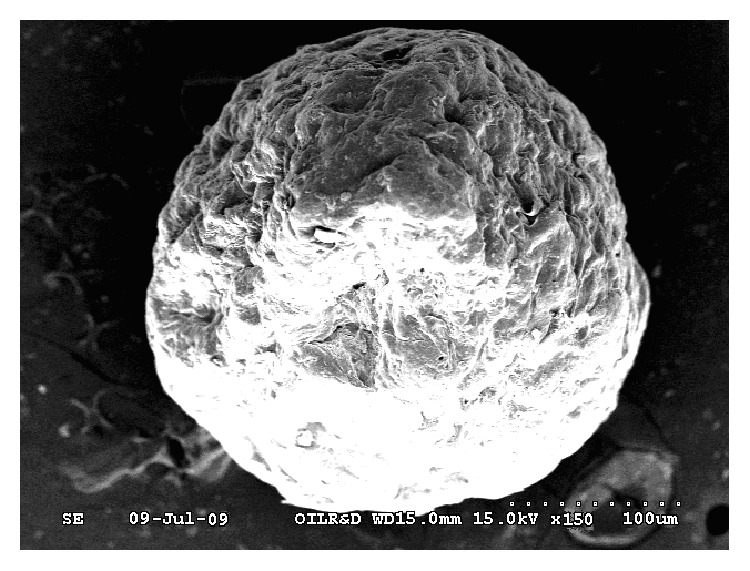
SEM image of metformin hydrochloride loaded microspheres (SD9) prepared using mucilage of* D. indica* by spray drying method at 150 magnification.

The mean particle sizes of the formulations were found to be in the range of 62 ± 6.24 to 98 ± 2.13 *μ*m in the formulations from SD1 to SD12. The particle size was found to be increased, when mucilage concentration was increased at constant inlet temperature, feed flow rate, and aspirator flow rate as observed in formulations SD3, SD6, SD9, and SD12 (Table 2). It was observed from the SEM photograph (Figure 2) of the samples of microspheres that the surfaces of the microspheres were rough with deepening on the surface. This was due to rapid loss of water from the surface on exposure to high temperature (Table 1). However, the microspheres were found to be almost spherical in shape.

### 4.2. Liquid Uptake Capacity

The liquid uptake capacity of microspheres was determined in water, phosphate buffer pH 7.4, and in 0.1 M HCl. The liquid uptake was found to be within the range of 10 ± 0.82 to 28 ± 0.14% in 0.1 M HCl; 16 ± 0.23 to 54 ± 0.13% in water, and 42 ± 1.07 to 66 ± 0.05% in phosphate buffer (pH 7.4). Maximum water uptake was observed in formulations containing higher amounts of the mucilage (SD3, SD6, SD9, and SD12) when inlet temperature, feed flow rate, and aspirator flow rate were kept constant (Tables 1 and 2). The uptake was low in 0.1 M HCl and in water but higher in phosphate buffer (pH 7.4). The acidic mucilage (pH 5.0) developed network structures in basic pH (pH 7.4) due to which more liquid uptake was observed in comparison to water and 0.1 M HCl.

### 4.3. Drug Entrapment Efficiency

The entrapment efficiency of different formulations is presented in Table 2. The entrapment efficiency was found to be in the range of 43.67 ± 0.58 to 55.12 ± 1.83 (SD1 to SD12). Highest entrapment efficiency was found to be 55.12 ± 1.83 (SF-12) in the formulation containing higher amount of the mucilage of* Dillenia indica*. The low entrapment efficiency was due to the aqueous solubility of MH and low water uptake capacity of the mucilage which in turn the drug was made available in the bulk solution before it was entrapped by the mucilage. The increased concentration of mucilage increased water uptake due to which more amount of the drug was available in the network structure of the mucilage. During spray drying process, the water evaporated leaving the drug within the mucilage network.

### 4.4. Mucoadhesive Property

**Figure 3:**
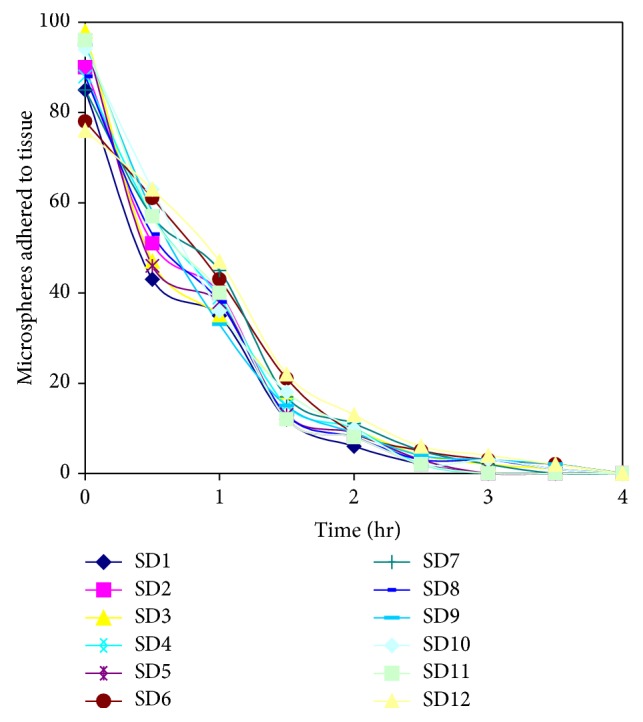
*In vitro* wash off test of metformin hydrochloride loaded microspheres prepared using mucilage of* D. indica* by spray drying method (SD1 to SD12).

The results of* in vitro* wash-off test of the spray dried microspheres prepared using the mucilage of* Dillenia indica* are presented in Figure 3. It was observed that the particles of formulations SD3, SD6, SD8, SD9, SD10, and SD12 remained adhered to the mucosal surface of the intestinal membrane up to 3.5 hrs.

The mucoadhesive property was found to be increased at increased concentration of mucilage in the formulations SD3, SD6, SD9, and SD12 (Table 1).

### 4.5. *In Vitro* Drug Release Study

**Figure 4:**
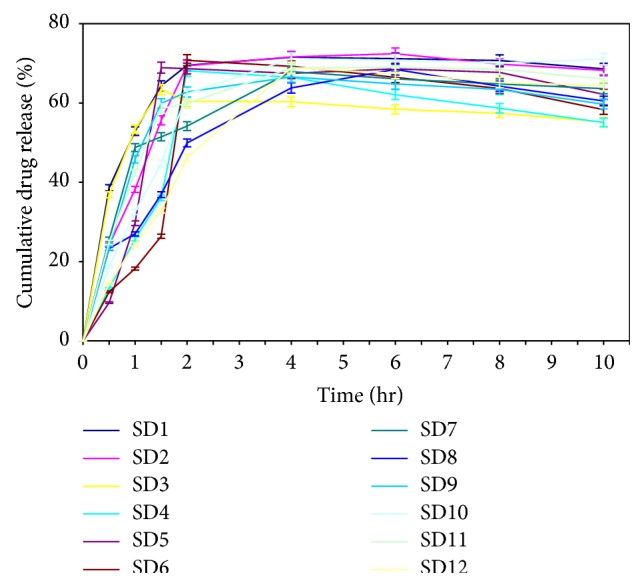
Cumulative percent drug release versus time profile of metformin hydrochloride loaded microspheres prepared using mucilage of* D. indica* by spray drying method (SD1 to SD12).

*In vitro* drug release study of the microspheres was carried out in phosphate buffer (pH 7.4) for 10 hrs. The results exhibited maximum drug release from most of the formulations within 4 hrs during the 10 hrs study except the formulations SD2, SD5, SD8, and SD11, which exhibited maximum drug release within 6 hrs during the 10 hrs study. These four formulations contained intermediate concentration of the mucilage (2.0 g) for a particular operating condition of spray dryer (Table 1). The* in vitro* drug release of the formulations was best explained by Higuchi kinetics with the highest linearity in formulations SD8, SD10, and SD12. The correlation coefficient (*R* ^2^) of the above formulations was 0.917, 0.863, and 0.909, respectively, which revealed that the drug diffused at a comparatively slower rate as the diffusional path length was increased. However, a direct correlation between the mucilage concentrations with the drug release kinetics could not be established due to the variation in the operating condition of the spray dryer. Moreover, the formulations also exhibited variations in the mechanism of drug release. Formulations SD1, SD2, SD3, SD7, SD8, SD9, and SD11 exhibited Fickian diffusion, whereas, SD4, SD5, SD6, SD10, and SD12 exhibited non-Fickian diffusion. This revealed that the mechanism was not directly related to the mucilage concentration but was related to the process parameters. The concentration versus time profile of the formulations (*Y* error = ±2%) is presented in Figure 4.

## 5. Conclusion

The microspheres prepared using the mucilage of DI by spray drying method exhibited poor entrapment efficiency but better mucoadhesive property. The microspheres remained adhered up to 3.5 hrs. The maximum liquid uptake was only 66.05%. The liquid uptake was the maximum in alkaline media and the minimum in 0.1 M HCl. Maximum amount of drug was released within 2 hrs and a decrease of concentration was observed during the study period of 10 hrs. However, there are options for improvement of the properties of microspheres for release of drug in a more controlled manner for a prolonged period of time.

## Figures and Tables

**Table 1:** It contains the composition and formulation parameters of metformin HCl loaded microspheres using mucilage of *D. indica* by spray drying method.

Formulation codes	Composition and formulation parameters					
	Mucilage of DI (%)	Inlet temp. (°C)	Outlet temp. (°C)	Feed flow rate (mL·min^−1^)	Aspirator flow rate (Nm^3^·mL^−1^)	Constant parameters
SD1	1	160	110	1.5	26	Metformin HCl (%) = 1.5; nozzle size (mm) = 0.7; pump speed (rpm) = 2.0; water (mL) = 200
SD2	2	160	110	1.5	26	
SD3	3	160	110	1.5	26	
SD4	1	170	115	1.7	27	
SD5	2	170	115	1.7	27	
SD6	3	170	115	1.7	27	
SD7	1	175	120	1.8	28	
SD8	2	175	120	1.8	28	
SD9	3	175	120	1.8	28	
SD10	1	180	130	1.9	29	
SD11	2	180	130	1.9	29	
SD12	3	180	130	1.9	29	

**Table 2:** *In vitro* evaluation of the metformin hydrochloride loaded spray dried microspheres prepared using mucilage of *D. indica*.

Formulation code	Mean particle size (*µ*m) ± SD; *n* = 80	Drug entrapment efficiency (%) ± SD; *n* = 3	Liquid uptake capacity in different media (%) ± SD; *n* = 3		
			0.1 M HCl	Water	Phosphate buffer (pH 7.4)
SD1	62 ± 4.09	43.67 ± 0.58	12 ± 1.22	26 ± 0.39	42 ± 1.07
SD2	69 ± 2.34	44.02 ± 1.05	16 ± 0.55	32 ± 0.60	44 ± 0.66
SD3	78 ± 4.24	47.05 ± 2.01	20 ± 0.74	48 ± 0.62	48 ± 0.23
SD4	76 ± 3.58	43.74 ± 1.24	10 ± 0.82	22 ± 0.22	42 ± 0.08
SD5	77 ± 1.08	45.13 ± 0.83	12 ± 0.19	16 ± 0.23	43 ± 0.26
SD6	85 ± 4.06	49.84 ± 1.68	28 ± 0.41	50 ± 0.39	54 ± 0.14
SD7	83 ± 3.34	44.78 ± 1.09	12 ± 0.34	42 ± 0.42	52 ± 0.22
SD8	84 ± 2.18	46.03 ± 2.01	28 ± 0.29	24 ± 0.37	57 ± 0.14
SD9	93 ± 3.87	53.17 ± 1.06	18 ± 0.25	52 ± 0.11	62 ± 0.92
SD10	78 ± 3.47	47.33 ± 1.57	17 ± 0.25	22 ± 0.53	48 ± 0.55
SD11	85 ± 2.11	52.73 ± 1.23	11 ± 0.24	18 ± 0.62	45 ± 0.64
SD12	98 ± 4.13	55.12 ± 1.83	28 ± 0.14	54 ± 0.13	66 ± 0.05
